# Addressing Healthy Aging: Time to Stop a Tsunami of Rising Alzheimer’s Disease

**DOI:** 10.14336/AD.2024.1476

**Published:** 2025-11-28

**Authors:** Nobel Chenggong Zong, Yuhan Zhang, Yuanli Huang, Hua Cai

**Affiliations:** Division of Molecular Medicine, Department of Anesthesiology and Perioperative Medicine, Division of Cardiology, Department of Medicine, David Geffen School of Medicine at University of California Los Angeles, California, 90095, USA

**Keywords:** Alzheimer’s disease (AD);, dementia, cognitive dysfunction, infection, artificial intelligence, CRISPR

## Abstract

Alzheimer’s disease [AD] disproportionately affects our seniors, diminishing their health and life expectancy. As the world population grows older, the collective burden of AD has become unsustainable. Globally, there were 43.8 million patients in 2016, with a projection of affecting 152 million by 2050. Recent discoveries have shown that molecular changes characteristic to AD manifested 20 years before discernable neurological phenotypes emerge. It is feasible to halt or reverse this pathological process before reaching an irremediable stage. To take advantage of this treatment window, we need to make rapid progress in early detection and monitoring, targeted implementation of preventative measures, invention of novel therapeutics, and pragmatic ramping-up of relevant supporting policies. PET is a powerful tool for prognosis. The utilization of AI technology, on the other hand, has favorable features of low cost per capita, easy dissemination and broad scale data collection to uncover previously unknown hotspots or risk factors. FDA approved drugs, lecanemab and donanemab, have started to show efficacy to put a pause on AD progression. Additional clinical data will enable comprehensive evaluation of the impacts of these drugs. Gene therapy holds the potential of eliciting long term protection, while several candidate loci have been identified. The urgency of a tsunami of rising AD epidemiology demands rapid actions on all fronts of advanced diagnostics, monitoring, preventative and interventive strategies.

Alzheimer’s disease (AD) accounts for 60-80% of dementia cases [1], with characteristic abnormal buildup of amyloid plaques and tau tangles. These changes interrupt communication between neurons, then microglia, chronic inflammation, disruption of glucose metabolism in the brain, followed by memory loss, cognitive decline, progressive loss in the ability to live independently, eventual bed-boundness and fatality four to eight years on average after diagnosis. There is no cure to date. For patients, this multiple year ordeal is not only physically debilitating, but also emotionally tormenting, seeing quality life slipping away yet not much can be done for reversal.

AD not only greatly handicaps healthy longevity of affected individuals, their family members offering care, but seniors altogether. Approximately 6.9 million Americans live with AD [2024] [1]; the affected is projected to reach 14 million by 2050, which would represent roughly 3% of the US population at that time. Globally, there were 43.8 million victims in 2016, with a projection of affecting 152 million by 2050 [2]. Despite great differences in age-structure, ethnicity, culture, economics, opportunities in education, healthcare availability, AD-related mortalities increased significantly across the globe [Fig. 1]. This is deteriorating since the population of our society is ageing, which represents the most significant risk factor for AD. For example, medical expenses for AD/dementia patients were estimated to rise from 345 billion in 2023 to 1 trillion USD in 2050, swallowing whole US Medicare budget into deficit [1]. The cost-insurance framework varies in other countries; nevertheless, seniors with AD generally need twice as many hospital-stays and more for hospice services, stretching already scant resources.

**Figure 1.**
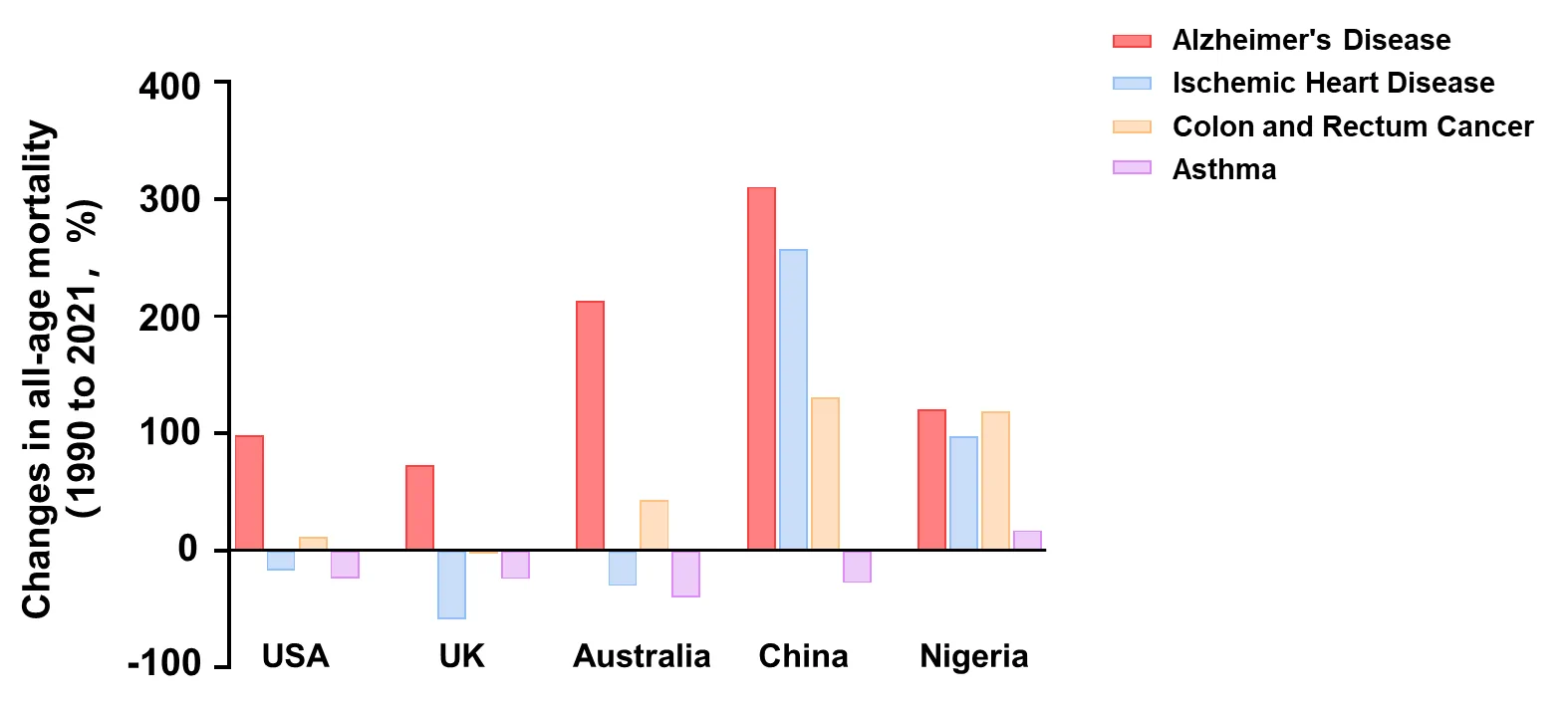
**A global perspective on changes in all-age mortality from 1990 to 2021**. During the 30-year span from 1990 to 2021, the burden of chronic disease-led mortality has changed significantly. The statistics of four major diseases, namely Alzheimer’s disease, ischemic heart disease, colon and rectum cancer, and asthma, were extracted from the The Global Burden of Disease (GBD) database and compared in five countries of USA (North America), UK (Europe), Australia (Oceania), China (Asia) and Nigeria (Africa). The numbers of mortality in 2021 were subtracted from that in 1990, and then divided by the numbers of mortality in 1990. AD-related mortality appears to be the most strikingly increased in each of these countries.

A silver lining emerged along the relative recent revelation that molecular-level changes begin 20 years ahead of neurological symptoms [3] of mild cognitive impairment [MCI] and AD [mild, moderate, severe]. MCI may still revert to asymptotic [4]. Innovations in prevention, diagnostics, therapeutics, and policy-reform are needed to take full advantage of this window of opportunity.

On prevention, a Lancet report [5] outlined fourteen modifiable risk factors, including obesity, diabetes, high cholesterol, hypertension, vision loss, hearing loss, smoking, excessive alcohol consumption, air pollution, head injury, depression, infrequent social contact, physical inactivity, and lower levels of education. Effective management of these risk factors could delay 40% of dementia cases [5]. In parallel, extended exposure to neurotoxins can drastically heighten the probability of developing AD-like illness, as evidenced by the impact of beta-methylaminoalanine on an island community of Guam [6].

Innovations in diagnostics will be pivotal for both preventative and interventive measures. At the molecular level, an amyloid PET scan can evaluate pathological accumulation of β-amyloid [7]; a FDG PET catches changes in glucose metabolism characteristic to AD. Medicare decision memos (CAG-00431R and 220.6.13) now offer PET scan coverage for seniors. Diagnosis will urge effective management of risk factors by seniors and their healthcare providers. At the neurological level, diagnosis of MCI is challenging for primary care physicians or geriatricians/neurologists, since physicians do not generally have frequent enough interactions with outpatients to identify an early cognitive decline. Artificial intelligence [AI] can come into aid. Monitoring daily usage patterns of smartphones may serve as a valuable tool. It can be programmed to keep log on usage patterns relevant to cognitive functions, such as the frequency of wrong password entries, the speed of keyboard inputs and typos, and numbers of occasions of getting lost. Furthermore, cognitive tests can be developed in APP format reminiscent to popular video games. The score matrix reflects short term memory and reaction time in decision making. The APP can be programmed to record a baseline and deviation from it for each user, capturing early changes sensitively. Moreover, APP is scalable for dissemination; thus, geologic hotspots or community groups with aberrant rate of AD can be revealed and investigated.

On medicinal treatment, US is leading the world with three FDA-approved drugs, aducanumab, lecanemab, and donanemab, which are tailored to molecular targets specific to AD. Biogen announced discontinuation of aducanumab in 2024. Lecanemab and donanemab are effective in approximately 30% of recipients with MCI or mild AD, slowing down cognitive decline by up to 8 months [8]. The annual price-tag is about 30 thousand dollars and roughly 30% of the recipients need to be monitored by professionals for brain swelling, a potentially serious complication. Taken together, these drugs are important addition to our toolbox; the gains over costs in budget and manpower remains to be audited from large-scale applications.

On another front of intervention, gene therapy, science has afforded us promising candidates. ApoE, as one example, has multiple isoforms. SNPs that distinguish these isoforms are well-known. The variations among amino acid residues at positions 112, 136 and 158 distinguish the three common variances: e2 (Cys, Arg, Cys), e3 (Cys, Arg, Arg) and e4 (Arg, Arg, Arg). Individuals inheriting dual copies of e4 isoform exhibiting 1,200% increase in AD incidents [1] over individuals with e2 isoform; and 56% of AD patients carry at least one copy of e4 isoform [9]. Replacing the Arg at 136 of e3 isoform with Ser, turning it into e3-Christchurch isoform which confers protective phenotype compared to the e3 isoform. Gene therapy, however, is a taboo for many not to trample on, which makes it a difficult topic for policymakers. Exceptional circumstances ask for courageous approaches. The world has demonstrated this capacity dealing with COVID-19 pandemic [10, 11] by approving vaccines and therapies at unprecedented pace.

On healthcare staffing, there is an ongoing shortage on geriatric specialists in the US (https://data.hrsa.gov/topics/health-workforce). This gap is going to expand further with a double whammy of a growing demand off an aging population, and a lowering interest for medical students and residents to choose geriatric medicine as a specialty [12]. The November 2023 National Resident Matching Program (NRMP) result showed geriatrics fellowship at the lowest fulfill rate across all specialties of medicine; yet among all internal medicine subspecialties, geriatricians have the lowest tendency to maintain their certification at 63% [13]. Besides delegating certain tasks to technology and nursing practitioners, boosting the capacities of geriatrics can be critically beneficial.

Alzheimer’s is complex in many ways. Although many distinct features have been documented, it remains unknown as to how and when this pathological process initially sets off. Autopsy report [14] showed that 82% of dementia cases are presented mixing AD with another type of dementia etiology (e.g., cerebrovascular disease, Parkinson’s). Current knowledge on AD is primarily gained from studies on the Caucasian population. The apparent distinction in vulnerable genes across ethnicities could be rooted from a variable mixing matrix of dementia with different causes. With broader application of PET scan on dementia patients, the contribution of various etiology will be better dissected. Thus, more specific and potentially integrated interventions may become necessary and beneficial. From a global perspective, the rate differences in AD prevalence increases (Fig. 1) cannot be fully attributed to variation in societal age structure. Finer probes into genetics and environmental factors can improve data interpretation.

No doubt, the population at risk of Alzheimer’s will increase spectacularly in the foreseeable next few decades. We need to ‘flatten the curve’, borrowing the popular slogan of the COVID-19 era. Innovations are needed in prevention, diagnostics, and therapeutics especially related to gene therapy. Thus, if emerging medications do not survive clinical trials in the next few years, society would at least have a mechanism to fall back on. Considering the chronicles of AD pathogenesis, we will reap the fruits of investments and policy-reforms 10-20 years later. Time is running short.
